# Children’s Hope in South Africa: A Population-Based Study

**DOI:** 10.3389/fpsyg.2020.01023

**Published:** 2020-06-03

**Authors:** Shazly Savahl

**Affiliations:** Child and Family Studies, University of the Western Cape, Bellville, South Africa

**Keywords:** children, hope, population-based study, confirmatory factor analysis, South Africa, Children’s Hope Scale

## Abstract

A growing body of research has provided evidence for the cognitive motivational construct of hope as a psychological strength, particularly for children in adverse social circumstances. In children, hope is defined as a set of cognitions focused on children’s agency to contemplate workable goals, to identify pathways to achieve those goals and the intrinsic beliefs about their capacity to activate sustained movement toward those goals. Using data from the third wave of the Children’s Worlds International Survey on Children’s Well-Being, the study aimed to explore children’s hope amongst a random population-based sample of children in South Africa. The study further aimed to explore children’s level of hope across the nine provincial regions of South Africa. Data were collected using Snyder et al.’s (1997) Children’s Hope Scale (CHS). Confirmatory factor analysis (CFA) was used to analyze the data, with multi-group CFA used to analyze the data across provincial regions. The study found an appropriate fit structure for the CHS using the overall pooled sample. The mean score on the CHS for the national sample was of 4.781 (*SD* = 1.082). Measurement invariance demonstrated the tenability of scalar invariance, which indicates comparability across correlations, regressions and mean scores. Mean scores ranged from 4.511 (*SD* = 1.163) for the Northern Cape to 4.982. (*SD* = 0.974) for the Western Cape. Five provinces (Eastern Cape, Northern Cape, Free State, Mpumalanga, and KwaZulu Natal) scored below the national mean, while four provinces (North West, Western Cape, Limpopo, and Gauteng) scored above.

## Introduction

A growing body of research has provided evidence for hope as a psychological strength, particularly for children and adolescents confronted with adverse conditions (Valle et al., 2006; Savahl et al., 2016). Empirical research oeuvres has established that hope is associated with subjective well-being, life satisfaction and overall quality of life among children and adolescents (e.g., Gilman et al., 2006; Sawyer et al., 2007; Marques et al., 2011; Merkaš and Brajša-Žganec, 2011; Martins et al., 2018; Raats et al., 2018). Within the social sciences, hope is conceptualized as a cognitive-motivational construct focusing on goal-directed behavior, the “future self,” and is encompassed in a typology of concepts, inclusive of coping, faith, resilience, and empowerment (Jevne, 1993).

Snyder’s theory on hope, developed across a period of more than 30-years, is the seminal theory in the field (Savahl et al., 2016). In describing Hope theory, Snyder (2002) delineated a cognitive model consisting of the “trilogy” of goals, pathways, and agency. “Goals” are the foundation of the theory and represents the cognitive component that grounds the theory. The theory works from the premise that people are goal-directed, with hope conceptualized as people’s perceptions regarding their capacities to (1) formulate clear goals, (2) develop the specific strategies or “workable routes” to reach those goals (pathways thinking), and (3) “self-related beliefs about initiating and sustaining movement toward those goals” (agentic thinking; Snyder et al., 1991, 1997, p. 401).

Both pathways and agency are fundamental in “hopeful thinking” and encompass relatively stable subjective evaluations of goal-oriented competencies (Snyder, 2000, 2002). The two components are positively correlated, additive, iterative, and reciprocal; however, they are not synonymous, and neither define hope separately (Snyder, 2002). In contrast to emotion-based theories of hope (see Farina et al., 1995), Snyder’s (2002) theoretical supposition of hope is decidedly cognitive and purports that individuals’ perceptions of goal pursuits are antecedent to, and drive emotions (see also Huebner, 1995; Ciarrochi et al., 2007, 2015). Those with high levels of hope are usually efficacious in their pursuit of goals, and typically, experience increased positive emotions (Snyder, 2002). In contrast, those with low levels of hope face additional challenges in attaining goals given various hindrances to goal attainment, and are likely to experience negative emotions (Snyder, 2002; Lopez et al., 2003).

In children, the theory portends that goal-directed hopeful thinking develops in the first few years of life and is crucial for the child’s development and survival (Snyder, 2002). How children make sense of and appraise their capacity to respond to challenges and barriers are important considerations for hope in children. Snyder et al. (1997) developed the Children’s Hope Scale (CHS) to address an identified gap in the literature in evaluating children’s hope and the key aspects that contribute to this construct. Diverging from earlier conceptualizations focusing on negative aspects of hope (see Kazdin et al., 1983), the scale emphasizes “positive expectancies.” The CHS is a six-item dispositional self-report scale developed for 8 to 16-year olds that evaluates the two key constituents of pathways and agency.

Along with the considerable reflection and engagement concerning conceptual definitions of hope, there has been an increasing investment in empirical studies exploring children’s hope using the CHS (Snyder et al., 1997, 2005) across various contexts. Within low and middle-income contexts, the work of Atik and Kemer (2009) in Turkey; Savahl et al. (2016), and Guse et al. (2016) in South Africa; Haroz et al. (2017) in Burundi, Indonesia, and Nepal; Wai et al. (2014) in Malaysia; Jovanoviæ (2013) in Serbia; and Lei et al. (2019) in China, have made substantial contributions to the literature on children’s hope. In high-income contexts, the empirical work of Ciarrochi et al. (2007, 2015) in Australia; Dixson (2017); Gilman et al. (2006); Dew-Reeves et al. (2012), and Valle et al. (2004, 2006) in the United States of America; Marques et al. (2009, 2014) in Portugal; Pulido-Martos et al. (2014) in Spain; and Wong and Lim (2009) in Singapore, are noteworthy. Taken together, all of the aforementioned studies delineate the CHS as a reliable and valid measure with samples of children and adolescents. These validation studies have revealed two different conceptualizations of the hope model. For example, while a two-factor model, as specified by the initial scale authors, was supported in some validation studies (see e.g., Valle et al., 2004; Pulido-Martos et al., 2014), others have found a better fit with a one-factor model (see e.g., Bickman et al., 2010; Dew-Reeves et al., 2012; Savahl et al., 2016).

While the focus of early research was on the validation of the CHS in various contexts, more recently the focus has shifted toward group comparisons and measurement invariance testing. For example, Lei et al. (2019) and Savahl et al. (2016) tested the measurement invariance of the CHS across socio-economic status groups, while Haroz et al. (2017) conducted group comparisons on samples of children in Burundi, Indonesia, and Nepal. Another recent trend in research is the relation of children’s hope with other psychological constructs such as quality of life (Martins et al., 2018), life satisfaction (Merkaš and Brajša-Žganec, 2011; Raats et al., 2018), and subjective well-being (Kaye-Tzadok et al., 2018). However, no studies have investigated hope using national representative or population-based samples of children, and standardized scores on the CHS have not been proposed. The goal of the present study is to explore hope in children using a nationally representative sample and to present a standardized mean score of the CHS.

## Aim of the Study

Using data from Wave 3 of the Children’s Worlds International Survey on Children’s Well-Being, the study aimed to explore hope amongst a random population-based sample of children in South Africa. The study further aimed to explore children’s level of hope across the nine provincial regions of South Africa.

## Method

### Research Design

The study forms part of Wave 3 of the Children’s Worlds International Survey on Children’s Well-Being^1^. Conducted across 35 countries, the survey is the largest multinational study to assess children’s subjective perceptions of their well-being across different contexts and domains. The study in South Africa employed a cross-sectional survey design and used a nationally representative proportionate stratified random sample of 10- and 12-year-olds, across the country’s nine provincial regions. A central management committee consisting of a range of experts in comparative international surveys was tasked with overseeing the sampling protocol, instrument development and data analytic plan of each participating country. It has been found that the central management of multinational collaborative studies leads to improved quality and integrity of the data (Casas and Rees, 2015).

### Participants and Sampling

The study included a nationally representative proportionate stratified random sample of children selected from public primary schools across the nine provincial regions of South Africa, namely: Western Cape; Eastern Cape; Northern Cape; North West; Mpumalanga; Gauteng; KwaZulu-Natal; Free State; and Limpopo. The stratification for the study was based on school grade (4 or 6), geographical context (urban or rural), and provincial region.

### Instrumentation

#### Children’s Hope Scale

The CHS developed by Snyder et al. (1997), measures goal-directed and hopeful thinking in children and adolescents between the ages of 8–16 years old. The scale consists of six items, with three questions evaluating “pathways thinking” (items 2, 4, and 6) and three evaluating “agency thinking” (items 1, 3, and 5). Response options are on a six-point verbal response format ranging from “None of the time” = 1 to “All of the time” = 6. A composite score is calculated by summing the raw scores on each item. Bickman et al. (2010) suggested mean cut-scores of low (<3.0), medium (3.0–4.67), and high (>4.67) hope categories, where higher scores are indicative of high levels of hope and lower scores indicate low levels of hope.

The CHS has shown good psychometric properties across a range of contexts (see the reliability analysis conducted by Hellman et al., 2018). Studies in South Africa conducted by Savahl et al. (2016) and Guse et al. (2016) have reported Cronbach’s alpha coefficients of 0.82 and 0.73, respectively.

### Data Analysis

Data were analyzed by means of confirmatory factor analysis (CFA; maximum likelihood estimation) using the Analysis of Moment Structures (AMOS, version 25) software. Following recommendations by Jackson et al. (2009), the Comparative Fit Index (CFI), Root Mean Squared Error of Approximation (RMSEA), and Standardized Root Mean Residual (SRMR) were used as fit indexes. Scores higher than 0.950 for the CFI and scores below 0.05 for the RMSEA and SRMR were regarded as a good fit. Improvement of model fit was achieved by the consideration of modification indices, standardized residual covariances, and the expected parameter change (Savahl et al., 2019). Measurement invariance was used to compare the results across provincial regions and was tested using multi-group confirmatory factor analysis (MGCFA) to ensure meaningful, reliable, and unambiguous group comparisons (Meredith, 1993; Millsap and Olivera-Aguilar, 2012). This process comprised three sequential steps wherein restrictive constraints were incrementally applied. In the first step, configural invariance was tested in a multi-group model by allowing the loadings and intercepts to be freely estimated; this represents the baseline model against which other models are tested. In the second step, metric invariance was tested by constraining the factor loadings. Finally, scalar invariance was tested by constraining the factor loadings and intercepts. Each subsequent constrained model was regarded as tenable if the model fit did not worsen by more than 0.01 on the CFI (Cheung and Rensvold, 2002) and by 0.015 on the RMSEA and SRMR (Chen, 2007). The tenability of scalar measurement invariance suggests that meaningful comparisons across groups (provincial regions) can be conducted by correlations, regression coefficients and mean scores. A means comparative analysis across the provincial regions was achieved through a one-way Analysis of Variance (ANOVA) using Stata (version 14).

### Procedure and Ethics

The South African study obtained ethics clearance from the Humanities and Social Sciences Research Ethics Committee of the University of the Western Cape, and the nine provincial education departments. Potential participants at each participating school attended a briefing session with the research team who explained the nature and details of the study. The research team also discussed the participants’ rights, the ethics principles of informed consent, confidentiality, the right to withdraw, privacy and the scientific use of the data. The final step in obtaining consent involved providing information sheets and consent forms to participants and seeking active consent from the children and their parents. The data collection process followed a researcher-administered protocol wherein the research team, led by the principal investigators, administered the questionnaire in a group setting to the participants by reading each question and explaining the response options. This took place during an administration period at the beginning of the school day with an average administration time of 30 minutes.

### Data Analytic Plan

The South African research team captured the data and submitted the database to the aforementioned central management committee, which oversaw the data management process. The initial database consisted of 7428 participants. The cleaning and depuration of the dataset followed a range of processes that included deleting cases with more than a third of missing data, the assessment of systematic response sets and attending to clustering due to survey design effects. The final dataset was weighted based on the proportion of children per provincial region. For the current study, analysis revealed missing items to be “missing completely at random.” Cases with two or less missing values on the CHS were substituted by regression imputation, as per the recommendations of Casas (2016). The final dataset consisted of 7067 participants (males = 45.6%, girls = 54.4%) between the ages of 9 to 12-years (*M*_age_ = 10.79, *SD* = 1.28), in Grades 4 (*n* = 3383), and 6 (*n* = 3684), attending 61 primary schools across the nine provincial regions of South Africa.

## Results

Skewness of the scores on the items of the CHS ranged from −1.222 to −0.916, with kurtosis ranging from −0.392 to 0.442. Given that these scores were outside the range of acceptable deviation, the bootstrap method (500 samples) in AMOS 25 was used as a resolution. A reliability analysis showed a Cronbach alpha of 0.80.

### Confirmatory Factor Analysis

**FIGURE 1:**
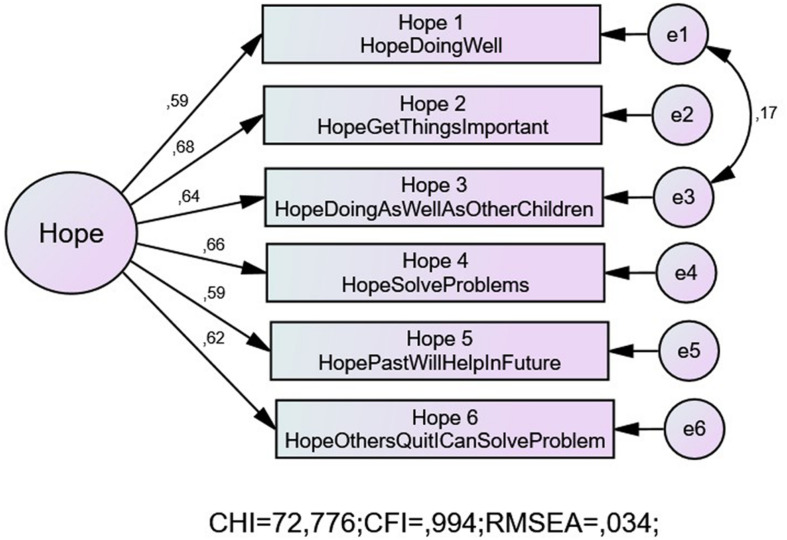
Modified model with one error covariance.

In line with the original scale authors’ supposition, a two-factor model was initially tested. However, while this model presented with an adequate fit, it showed an unacceptably high correlation between the latent constructs (0.92). This suggests that the two constructs are indistinguishable and would likely lead to convergent validity issues. Thereafter, a single factor model was tested; this model did not meet the criteria for an adequate fit. However, through the consideration of the modification indices, an error covariance was included between Item 1 (I think I am doing well) and Item 3 (I am doing just as well as other children my age). This resulted in an excellent fit (see Model 3 in Table 1 and Figure 1). Standardized regression weights ranged from 0.59 to 0.68 and were all significant at the 0.001 level (see Figure 1).

**TABLE 1:** Fit indexes for the confirmatory factor models.

Model Bootstrap, ML, 95% Confidence Intervals, Resamples = 500	Chi-Square	*df*	*p*-value	CFI	RMSEA	SRMR
1. Initial two-factor model	123.566	8	0.000	0.989	0.045 (0.038–0.052)	0.0182
2. Initial one-factor model	200.387	9	0.000	0.982	0.055 (0.048–.062)	0.0223
3. Modified one-factor model with one error covariance	72.776	8	0.000	0.994	0.034 (0.027–.041)	0.0138
4. Configural one-factor model (across provincial region)	186.548	72	0.000	0.989	0.015 (0.012–.018)	0.0185
5. Metric one-factor model (across provincial region)	295.001	112	0.000	0.986	0.015 (0.013–.017)	0.0263
6. Scalar one-factor model (across provincial region)	377.232	152	0.000	0.979	0.014 (0.013–.016)	0.0363

### Multi-Group Confirmatory Factor Analysis

As previously mentioned, measurement invariance across provincial regions was tested using MGCFA through a sequential process of applying increasingly restrictive constraints. Given that each subsequent constrained model did not worsen by more than 0.01 on the CFI, nor by 0.015 on the RMSEA and SRMR (Cheung and Rensvold, 2002; Chen, 2007) configural, metric, and scalar measurement invariance was attained (see Models 4–6). This means that across provincial regions, children’s scores on the hope scale can be compared by correlations, regression coefficients, and means. The standardized regression weights (constrained loadings and intercepts) which represents the scalar measurement model, are presented in Table 2.

**TABLE 2:** Standardized regression weights: (Provincial regions – constrained loadings and intercepts).

Parameter			Eastern Cape			North West			Western Cape		
			Estimate	Lower	Upper	Estimate	Lower	Upper	Estimate	Lower	Upper
Hope doing well	<–	Hope	0.616	0.579	0.656	0.565	0.516	0.615	0.571	0.524	0.613
Hope get things important	<–	Hope	0.704	0.664	0.742	0.651	0.602	0.702	0.674	0.625	0.722
Hope doing as well as other children	<–	Hope	0.651	0.610	0.690	0.598	0.548	0.649	0.641	0.598	0.680
Hope solve problems	<–	Hope	0.645	0.603	0.682	0.577	0.528	0.627	0.662	0.615	0.708
Hope past will help in future	<–	Hope	0.625	0.585	0.660	0.550	0.504	0.606	0.571	0.522	0.616
Hope others quit i can solve problem	<–	Hope	0.616	0.585	0.646	0.605	0.551	0.659	0.611	0.564	0.657

			**Northern Cape**			**Free State**			**Mpumalanga**		
			**Estimate**	**Lower**	**Upper**	**Estimate**	**Lower**	**Upper**	**Estimate**	**Lower**	**Upper**

Hope doing well	<–	Hope	0.545	0.477	0.612	0.552	0.492	0.612	0.599	0.553	0.658
Hope get things important	<–	Hope	0.704	0.620	0.782	0.684	0.621	0.752	0.636	0.575	0.691
Hope doing as well as other children	<–	Hope	0.701	0.626	0.775	0.634	0.574	0.698	0.632	0.576	0.686
Hope solve problems	<–	Hope	0.699	0.625	0.781	0.644	0.580	0.713	0.677	0.627	0.726
Hope past will help in future	<–	Hope	0.582	0.513	0.657	0.524	0.462	0.586	0.577	0.519	0.630
Hope others quit i can solve problem	<–	Hope	0.616	0.539	0.689	0.619	0.562	0.683	0.594	0.541	0.645

			**Limpopo**			**Gauteng**			**KwaZulu Natal**		
			**Estimate**	**Lower**	**Upper**	**Estimate**	**Lower**	**Upper**	**Estimate**	**Lower**	**Upper**

Hope doing well	<–	Hope	0.608	0.574	0.644	0.622	0.585	0.656	0.565	0.535	0.600
Hope get things important	<–	Hope	0.693	0.653	0.730	0.689	0.650	724	0.648	0.616	0.680
Hope doing as well as other children	<–	Hope	0.631	0.590	0.671	0.660	0.622	0.694	0.604	0.572	0.633
Hope solve problems	<–	Hope	0.676	0.635	0.715	0.691	0.651	0.724	0.633	0.601	0.666
Hope past will help in future	<–	Hope	0.623	0.587	0.659	0.609	0.567	0.652	0.561	0.530	0.590
Hope others quit i can solve problem	<–	Hope	0.631	0.594	0.668	0.653	0.614	0.691	0.581	0.548	0.613

**All values are significant at <.001.**

### Comparisons Across Mean Scores

The mean score of the pooled sample using the weighted data was *M* = 4.781 (*SD* = 1.082). Given the tenability of scalar measurement invariance across provincial regions, a comparative means analysis was apposite. A one-way ANOVA demonstrated significant mean differences between the provincial regions [*F* = 22.981; *df* = 8; *p* < 0.001; η^2^ = 0.025; and 95% CI = (0.0178, 0.0320)]. Mean scores ranged from 4.511 (*SD* = 1.163) for the Northern Cape to 4.982. (*SD* = 0.974) for the Western Cape (see Table 3). Five provinces (Northern Cape, Eastern Cape, Mpumalanga, KwaZulu Natal, and the Free State) scored below the national mean, while four provinces (North West, Western Cape, Limpopo, and Gauteng) scored above. Using the threshold cut-scores proposed by Bickman et al. (2010), two provinces scored within the category of “medium-hope,” while seven provinces scored within the “high-hope” category (see Table 3). Table 3 also presents the percentage of the overall and provincial samples scoring below the overall country mean. For the overall country sample, 41.9% scored below the mean, with percentages ranging from 33.2 % for the North-West Province to 52.7% for the Northern Cape.

**TABLE 3:** Mean scores and percentage of the sample scoring below the overall mean.

Provincial Region	*N*	Mean	*SD*	% scoring below overall country Mean
Eastern Cape	988	4.723	1.077	44.4
North West	627	4.972	0.952	33.2
Western Cape	734	4.982	0.974	35.1
*Northern Cape	201	4.511	1.163	52.7
Free State	279	4.708	1.123	44.8
Mpumalanga	486	4.702	1.001	48.4
Limpopo	1195	4.958	1.086	34.2
Gauteng	1145	4.841	1.126	48.9
*Kwa-Zulu Natal	1412	4.520	1.096	51.9
Overall	7067	4.781	1.082	41.9

*These provinces score within the medium-hope category. All other provinces score within the high-hope category.

## Discussion

Using data from Wave 3 of the Children’s Worlds: International Survey on Children’s Well-Being, this study aimed to explore hope amongst a random population-based sample of children in South Africa. The study further aimed to explore children’s level of hope across the nine provincial regions. CFA demonstrated an appropriate fit structure for the CHS using a population-based sample of children, while MGCFA confirmed the tenability of scalar measurement invariance.

Confirmatory factor analysis found that while a two-factor model of the CHS presented with an appropriate fit, an unacceptably high correlation was observed between the latent constructs (pathways and agency). This calls into question the distinctiveness of the latent constructs and could result in convergent validity issues. This finding resonates with previous research conducted by Savahl et al. (2016) who found similarly high correlations between the latent constructs in a South African sample. A one-factor model presented with an appropriate fit structure with the addition of one error co-variance. In later commentary, one of the original scale authors acknowledged the supposition of a one-factor model and recommended further exploration of the unidimensional structure (Lopez et al., 2000). The inclusion of the correlated error was justified based on probable method bias and is likely due to the semantic overlap of the content of the items. Standardized regression weights were all acceptable, ranging from 0.59 to 0.68. The findings ultimately indicate an appropriate fit structure for the overall pooled sample. The mean score for the overall pooled sample (*M* = 4.781, *SD* = 1.082) can be categorized as “high hope” according to the cut-scores proposed by Bickman et al. (2010). Given the use of a randomized population-based sample, this mean score represents a standardized score and is generalizable to the population of 10- to 12-year-old children attending public schools in South Africa.

Multi-group confirmatory factor analysis was used to test the comparability of the CHS across provincial regions. Given that scalar invariance was tenable, the scores on the CHS across provincial regions can be meaningfully compared by correlations, regression coefficients, and means. The results show significant mean differences between provincial regions, with the Western Cape, North West, Limpopo, and Gauteng scoring above the national mean; while the Northern Cape, Eastern Cape, Mpumalanga, KwaZulu Natal, and the Free State scored below the national mean. Furthermore, the Northern Cape and KwaZulu Natal were the only two provinces that scored in the “medium hope” category, while the other provinces all scored within the “high hope” category. Interestingly, the Western Cape, the province with the highest human development index in South Africa (United Nations Development Programme, 2003; Western Cape Government, 2005) presented with the highest mean score on the CHS (*M* = 4.982, *SD* = 0.974).

## Conclusion

This is the first study to use a nationally representative sample to measure hope in children in South Africa. Given the use of a randomized sample, and the attainment of an appropriate fit structure for the CHS, the overall mean score represents a standardized score of 10- to 12-year-old children attending public schools in South Africa. The results from the study suggest that children in South Africa present with high levels of hope. This finding is important given the often-maligned social conditions and constrained socio-economic contextual realities associated with growing up in South Africa. With the construct of hope being closely related to subjective well-being and quality of life (Raats et al., 2018) the study highlights the need for further research to understand the factors related to hope. To this end, longitudinal research could provide a more complete understanding of the mechanisms through which these factors function. Future research should also endeavor to explore the measurement invariance of hope across other groups or cohorts of the child population. Finally, given the role of hope as causal to emotions, self-esteem and other psychological constructs and behaviors, social service and educational practitioners should focus their efforts on developing goal-directed thinking in children.

## Data Availability Statement

The datasets generated for this study are available on request to the corresponding author.

## Ethics Statement

The studies involving human participants were reviewed and approved by Humanities and Social Sciences Research Ethics Committee of the University of the Western Cape. Written informed consent to participate in this study was provided by the participants and their parents/legal guardians.

## Author Contributions

SS conceptualized and executed the study.

## Conflict of Interest

The author declares that the research was conducted in the absence of any commercial or financial relationships that could be construed as a potential conflict of interest.

## Footnotes

1 www.isciweb.org

## References

[B1] Atik G. Kemer G. (2009). Psychometric properties of Children’s hope scale: validity and reliability study. *Element. Educ. Online* 8 379–390.

[B2] Bickman L. Athay M. M. Riemer M. Lambert E. W. Kelley S. D. Breda C., et al. (eds) (2010). *Manual of the Peabody Treatment Progress Battery*, 2nd Edn, Nashville: Vanderbilt University.

[B3] Casas F. (2016). Analysing the comparability of 3 multi-item subjective well-being psychometric scales among 15 countries using samples of 10 and 12-year-olds. *Child Indic. Res.* 10 297–330. 10.1007/s12187-015-9360-0

[B4] Casas F. Rees G. (2015). Measures of children’s subjective well-being: analysis of the potential for cross-national comparisons. *Child Indic. Res.* 8 49–69. 10.1007/s12187-014-9293-z

[B5] Chen F. F. (2007). Sensitivity of goodness of fit indexes to lack of measurement invariance. *Struct. Equ. Model.* 14 464–504. 10.1080/10705510701301834

[B6] Cheung G. W. Rensvold R. B. (2002). Evaluating goodness-of-fit indexes for testing MI. *Struct. Equ. Model.* 9 235–255.

[B7] Ciarrochi J. Heaven P. C. Davies F. (2007). The impact of hope, self-esteem, and attributional style on adolescents’ school grades and emotional well-being: a longitudinal study. *J. Res. Pers.* 41 1161–1178. 10.1016/j.jrp.2007.02.001

[B8] Ciarrochi J. Parker P. Kashdan T. B. Heaven P. C. L. Barkus E. (2015). Hope and emotional well-being: a six-year study to distinguish antecedents, correlates, and Consequences. *J. Posit. Psychol.* 10 520–532. 10.1080/17439760.2015.1015154

[B9] Dew-Reeves S. E. Athay M. M. Kelley S. D. (2012). Validation and use of the Children’s hope scale-revised PTPB edition (CHS-PTPB): high initial youth hope and elevated baseline symptomatology predict poor treatment outcomes. *Adm. Policy. Ment. Health* 39 60–70. 10.1007/s10488-012-0411-2 10.1007/s10488-012-0411-2 22407561

[B10] Dixson D. D. (2017). Hope across achievement: examining psychometric properties of the Children’s Hope Scale across the range of achievement. *SAGE Open* 7 1–11. 10.3389/fpsyg.2019.02593 31824379 PMC6881258

[B11] Farina C. J. Hearth A. K. Popovich J. M. (1995). *Hope and Hopelessness: Critical Clinical Constructs.* Thousand Oaks, CA: Sage.

[B12] Gilman R. Dooley J. Florell D. (2006). Relative levels of hope and their relationship with academic and psychological indicators among adolescents. *J. Soc. Clin. Psychol.* 25 166–178. 10.1521/jscp.2006.25.2.166

[B13] Guse T. de Bruin G. P. Kok M. (2016). Validation of the children’s hope scale in a sample of south african adolescents. *Child Indic. Res.* 9 757–770. 10.1007/s12187-015-9345-z

[B14] Haroz E. E. Jordans M. de Jong J. Gross A. Bass J. Tol W. (2017). Measuring hope among children affected by armed conflict: cross-cultural construct validity of the children’s hope scale. *Assessment* 24 528–539. 10.1177/1073191115612924 26508802 PMC5835958

[B15] Hellman C. M. Munoz R. T. Worley J. A. Feeley J. A. Gillert J. E. (2018). A reliability generalization on the Children’s Hope Scale. *Child Indic. Res.* 11 1193–1200. 10.1007/s12187-017-9467-6

[B16] Huebner E. S. (1995). The students’ life satisfaction scale: an assessment of psychometric properties with black and white elementary school students. *Soc. Indic. Res.* 34 315–323. 10.1007/bf01078690

[B17] Jackson D. L. Gillaspy J. A. Jr. Purc-Stephenson R. (2009). Reporting practices in confirmatory factor analyses: an overview and some recommendations. *Psychol. Methods* 14 6–23. 10.1037/a0014694 19271845

[B48] Jevne R. F. (1992). “Enhancing hope in chronically ill in the national Institutes of Health, Immunity, and Disease,” in *Clinical Approaches To Behavioural Medicine: The Healing Methods Of A New Generation*, (Mansfield, CT: Mansfield Center), 127–132.

[B18] Jevne R. (1993). Enhancing hope in the chronically ill. *Hum. Med.* 9, 121–130.

[B19] Jovanoviæ V. (2013). Evaluation of the Children’s Hope scale in serbian adolescents: dimensionality, measurement invariance across gender, convergent and incremental validity. *Child Indic. Res.* 6 797–811. 10.1007/s12187-013-9195-5

[B20] Kaye-Tzadok A. Ben-Arieh A. Kosher H. (2018). Hope, material resources, and subjective well-being of 8- to 12-year-old children in Israel. *Child Dev.* 90 344–358. 10.1111/cdev.13130 30125932

[B21] Kazdin A. E. French N. H. Unis A. S. Esveldt-Dawson K. Sherick R. B. (1983). Hopelessness, depression, and suicidal intent among psychiatrically disturbed inpatient children. *J. Consult. Clin. Psychol.* 51 504–510. 10.1037/0022-006X.51.4.504 6619356

[B22] Lei H. Wang Z. Peng Z. Yuan Y. Li Z. (2019). Hope across socioeconomic status: examining measurement invariance of the Children’s hope scale across socioeconomic status groups. *Front. Psychol.* 21:2593. 10.3389/fpsyg.2019.02593 31824379 PMC6881258

[B23] Lopez S. J. Gariglietti K. P. McDermott D. Sherwin E. D. Floyd R. K. Rand K. (2000). “Hope for the evolution of diversity: on leveling the field of dreams,” in *Handbook of Hope: Theory, Measures And Applications*, ed. Snyder C. R. (California: Academic Press), 223–240.

[B24] Lopez S. J. Snyder C. R. Pedrotti J. T. (2003). “Hope: Many definitions, many measures,” in *Positive Psychological Assessment: A Handbook Of Models And Measures*, eds Lopez S. J. Snyder C. R. (Washington, DC: American Psychological Association), 91–106. 10.1037/10612-006

[B25] Marques S. C. Lopez S. J. Fontaine A. M. Coimbra S. Mitchell J. (2014). Validation of a Portuguese version of the Snyder Hope scale in a sample of high school students. *J. Psychoeduc. Assessm.* 32 781–786. 10.1177/0734282914540865

[B26] Marques S. C. Lopez S. J. Pais-Ribeiro J. L. (2011). Building hope for the future: a program to foster strengths in middle-school students. *J. Happiness Stud.* 12 139–152. 10.1007/s10902-009-9180-3

[B27] Marques S. C. Pais-Ribeiro J. L. Lopez S. J. (2009). Validation of a portuguese version of the Children’s Hope Scale. *Sch. Psychol. Intern.* 30 538–551. 10.1177/0143034309107069

[B28] Martins A. R. Crespo C. Salvador A. Santos S. Carona C. Canavarro M. C. (2018). Does hope matter? Associations among self-reported hope, anxiety, and health-related quality of life in children and adolescents with cancer. *J. Clin. Psychol. Med. Settings* 25 93–103. 10.1007/s10880-018-9547-x 29453505

[B29] Meredith W. (1993). Measurement invariance, factor analysis, and factorial invariance. *Psychometrika* 58 525–542. 10.1007/s11136-013-0465-y 23836434

[B30] Merkaš M. Brajša-Žganec A. (2011). Children with different levels of hope: are there differences in their self-esteem, life satisfaction, social support, and family cohesion? *Child Indic. Res.* 4 499–514. 10.1007/s12187-011-9105-7

[B31] Millsap R. E. Olivera-Aguilar M. (2012). “Investigating measurement invariance using confirmatory factor analysis,” in *Handbook of Structural Equation Modeling*, ed. Hoyle R. H. (New York, NY: Guilford), 380–392. 10.1017/S0033291718000399

[B32] Pulido-Martos M. Jiménez-Moral J. A. Lopez-Zafra E. Ruiz J. R. (2014). An adaptation of the Children’s Hope Scale in a sample of Spanish adolescents. *Child Indic. Res.* 7 267–278. 10.1007/s12187-013-9223-5

[B33] Raats C. Adams S. Savahl S. Isaacs S. A. Tiliouine H. (2018). The relationship between hope and life satisfaction among children in low and middle socio-economic status communities in Cape Town, South Africa. *Child Indic. Res.* 12 1–14.

[B34] Savahl S. Adams S. Florence M. Casas F. Mpilo M. Isobell D. (2019). The relation between children’s participation in daily activities, their engagement with family and friends, and subjective well-being. *Child Ind. Res.* 10.1007/s12187-019-09699-3

[B35] Savahl S. Casas F. Adams S. (2016). Validation of the Children’s Hope Scale amongst a sample of adolescents in the Western Cape region of South Africa. *Child Indic. Res.* 9 701–713. 10.1007/s12187-015-9334-2

[B36] Sawyer W. Singh M. Woodrow C. Downes T. Johnston C. Whitton D. (2007). Robust hope and teacher education policy. *Asia Pac. J. Teach. Educ.* 35 227–242. 10.1080/13598660701447197

[B37] Snyder C. R. (2000). The past and future of hope. *J. Soc. Clin. Psychol.* 19 11–28.

[B38] Snyder C. R. (2002). Hope theory: rainbows in the mind. *Psychol. Inq.* 13 249–275. 10.1207/s15327965pli1304_01

[B39] Snyder C. R. Hoza B. Pelham W. E. Rapoff M. Ware L. Danovsky M. (1997). The development and validation of the Children’s Hope Scale. *J. Pediatr. Psychol.* 22 399–421. 10.1093/jpepsy/22.3.399 9212556

[B40] Snyder C. R. Irving L. Anderson J. R. (1991). “Hope and health: measuring the will and the ways,” in *Handbook of Social And Clinical Psychology: The Health Perspective*, eds Snyder C. R. Forsyth D. R. (Elmsford, NY: Pergamon Press), 285–305.

[B41] Snyder C. R. Rand K. L. Sigmon D. R. (2005). “Hope theory: a member of the positive psychology family,” in *Handbook of Positive Psychology*, eds Snyder C. R. Lopez S. J. (New York, NY: Oxford University Press), 257–267.

[B42] United Nations Development Programme (2003). *South Africa Human Development Report, The Challenge Of Sustainable Development in South Africa: Unlocking People’s Creativity.* Oxford: Oxford University Press.

[B43] Valle M. F. Huebner E. S. Suldo S. M. (2004). Further evaluation of the children’s hope scale. *J. Psychoeduc. Assess.* 22 320–337. 10.1177/073428290402200403

[B44] Valle M. F. Huebner E. S. Suldo S. M. (2006). An analysis of hope as a psychological strength. *J. Sch. Psychol.* 44 393–406. 10.1016/j.jsp.2006.03.005

[B45] Wai C. M. Talib M. A. Yaacob S. N. Jo-Pei T. Awang H. Hassan S. (2014). Hope and its relation to suicidal risk behaviors among Malaysian adolescents. *Asian Soc. Sci.* 10 67–71.

[B46] Western Cape Government (2005). *Overberg District Municipality Spatial Development Framework.* Cape Town: Western Cape Government.

[B47] Wong S. S. Lim T. (2009). Hope versus optimism in Singaporean adolescents: contributions to depression and life satisfaction. *Pers. Individ. Differ.* 46 648–652. 10.1016/j.paid.2009.01.009

